# Protective Effect of Chinese Bayberry (*Myrica rubra* Sieb. et Zucc.) Pomace Wine on Oxidative Stress of Hydrogen Peroxide by Regulating Keap1/Nrf2 Pathway in HepG2 Cells

**DOI:** 10.3390/foods12091863

**Published:** 2023-04-30

**Authors:** Jing Jiang, Yanyun Zhu, Mengting Wang, Jianchu Chen

**Affiliations:** 1 National-Local Joint Engineering Laboratory of Intelligent Food Technology and Equipment, Zhejiang Key Laboratory for Agro-Food Processing, Zhejiang Engineering Laboratory of Food Technology and Equipment, College of Biosystems Engineering and Food Science, Zhejiang University, Hangzhou 310058, China; 2 Zhejiang University Zhongyuan Institute, Zhengzhou 450001, China; 3 School of Biological and Chemical Engineering, NingboTech University, Ningbo 315100, China

**Keywords:** Chinese bayberry pomace wine, antioxidant activity, oxidative damage, Keap1/Nrf2 pathway

## Abstract

Chinese bayberry (*Myrica rubra* Sieb. et Zucc.) pomace wine (CPW) is fruity and rich in bioactive compounds, with high nutritional value and antioxidant activities. This study aims to investigate the protective effect of CPW on the oxidative damage induced by hydrogen peroxide in human hepatocellular carcinoma (HepG2) cells and CPW’s possible underlying mechanism. The fluorescence assay results revealed that CPW pre-treatment inhibited intracellular reactive oxygen species (ROS) accumulation in H_2_O_2_-induced HepG2 cells and cell membrane injury. Meanwhile, CPW remarkably enhanced the activity of antioxidant enzymes including superoxide dismutase (SOD), catalase (CAT), glutathione reductase (GR), and the content of glutathione (GSH). Moreover, CPW pretreatment significantly regulated the expression of Kelch-like ECH-associated protein 1 (Keap1)/nuclear factor erythroid 2-related factor 2 (Nrf2) pathway-associated genes (*Keap1, Nrf2, NADPH quinone oxidoreductase I* (*NQO1*)*,* and *heme oxygenase-1* (*HO-1)*) and its downstream antioxidant genes (*SOD, CAT, GSH*, and the *glutamate-cysteine ligase catalytic* (*GCLC*) *subunit*) in HepG2 cells. These data demonstrated that CPW prevented H_2_O_2_-induced oxidative stress by regulating the Keap1/Nrf2 signaling pathway.

## 1. Introduction

Chinese bayberry (*Myrica rubra* Sieb. et Zucc.) pomace is a by-product of bayberry juice production. Research on the fermentation of the discarded bayberry pomace with yeast has a positive significance for promoting the sustainable development of the bayberry juice industry and the reasonable usage of natural resources. Fermentation can enhance the nutritional value of fruits and increase the antioxidant activity of several natural products [1]. Co-fermentation of Chinese bayberry pomace using saccharomycetes, lactic acid bacteria, and ethylic acid bacteria could improve the appearance, the color parameters, and the content of the phenolic acids of Chinese bayberry pomace beverages [2]. A previous study found that the polysaccharides from Chinese bayberry pomace wine could scavenge 1,1-diphenyl-2-picrylhydrazyl (DPPH) and 2,2′-azino-bis(3-ethylbenzothiazoline-6-sulfonate) (ABTS^+^) radicals and inhibit the proliferation of HepG2 cells [3]. Growing evidence suggests that diets rich in antioxidant phytochemicals, including polyphenolic compounds, polysaccharides, tocopherols, and anthocyanins, can protect the body from free radicals and have potentially beneficial effects on human health [4,5,6]. Therefore, CPW has great market prospects owing to its rich bioactive substances, high nutritional value, and antioxidant activities.

Oxidative stress means a steady-state unbalance between ROS generation and the antioxidative defense mechanisms in an organism, which can lead to cell functional disorder, DNA lesions, potential genetic mutations, and finally apoptosis [7,8,9]. ROS are produced during mitochondrial oxidative phosphorylation [10]. According to epidemiological studies and clinical research, oxidative stress caused by free radicals gives rise to numerous chronic diseases, which are associated with the pathological process of cancer and cardiovascular and cerebrovascular diseases [11,12,13,14]. To date, chemical methods in vitro have been most extensively used to evaluate the antioxidant activity of chemical compounds against oxidative stress. Nevertheless, antioxidation is not only confined to ROS clearance but also involves the upregulation of antioxidant and detoxifying enzymes, regulations of cell signal transduction and genetic expression, and other cellular effects [15]. On account of its better relevance with biological systems, cell-based methods in vivo can overcome the boundedness of chemical approaches. It is widely accepted to use HepG2 cells as a model to estimate the antioxidant activity and investigate the potential mechanisms of HepG2 cells against various oxidative stresses.

In response to external oxidative stress, organisms have evolved many defense systems to clear away stimuli. The Kelch-like ECH-associated protein 1 (Keap1)/nuclear factor erythroid 2-related factor 2 (Nrf2) system is one of the most essential cellular defense systems, responsible for activating the activity of antioxidant enzymes under oxidative stress in vivo [16,17,18]. Wang et al. suggested that apple phlorizin reversed the decrease in the antioxidative enzymes CAT and SOD and the glutathione peroxidase (GPx) in H_2_O_2-_induced HepG2 cells by activating the Nrf2 signaling pathway [5]. Li et al. proposed that neuroligin-3 inhibited cell apoptosis by activating the Nrf2 signaling and promoting the expression of the important antioxidant enzymes (HO-1, GCLC, and NQO1) induced by H_2_O_2_ in retinal cells [19]. In addition, the peptide from porcine plasma hydrolysates can scavenge ROS and malondialdehyde (MDA) to enhance the effect of the Keap1-Nrf2-antioxidant response element (ARE) signaling pathway in HepG2 cells, thus enhancing the ability of cells to protect against oxidant stress [20]. Consequently, we can focus on the Keap1/Nrf2 pathway to study the antioxidant mechanism of CPW.

Previous studies have shown that CPW has important natural bioactive substances with diverse benefits, mainly in antioxidation. To our knowledge, however, the protective effect of CPW against H_2_O_2_-induced oxidative stress is yet to be clarified. In general, the present study is intended to investigate the intracellular antioxidant capacities of CPW on a H_2_O_2_-induced oxidative damage model in HepG2 cells, including cell cytotoxicity, cell apoptosis, the intracellular ROS level, antioxidant enzyme activity, and the expression of antioxidant-related genes. This study excavates the possible antioxidant mechanisms of CPW from the cellular level, which is conducive to subsequent in vivo research and application.

## 2. Material and Methods

### 2.1. Chemicals and Reagents

DPPH was purchased from Shanghai Chemical Reagent Co. (Shanghai, China); ABTS was purchased from Hefei Bomei Biotechnology Co., Ltd. (Hefei, China); Dulbecco’s Modified Eagle’s Medium (DMEM), trypsin-EDTA (0.25%), fetal bovine serum (FBS), 3-(4,5-dimethylthiazol-2-yl)-2,5-diphenyltetrazolium bromide (MTT) and penicillin–streptomycin were bought from Gibco, Life Technologies (Grand Island, NY, USA). Radio immunoprecipitation assay (RIPA) buffer, BCA protein assay kit, SOD, MDA, GPx, oxidized glutathione (GSSG) assay kits, and total antioxidant capacity assay kit with ferric-reducing ability of plasma (FRAP) method were all obtained from Beyotime Biotechnology (Shanghai, China). CAT assay kit and GR assay kit were bought from Solarbio Science & Technology (Beijing, China). 2′,7′-Dichlorofluorescein diacetate (DCFH-DA) and propidium iodide (PI) were purchased from Sigma Chemical Company (St. Louis, MO, USA). TRIGene regent was bought from GenStar (Beijing, China). PrimeScript™ RT reagent kit with gDNA eraser was bought from Takara (Dalian, China). Power SYBR green master mix was bought from Thermo Fisher Scientific (Wilmington, DE, USA).

### 2.2. Materials and Microbial Strains

The Biqi bayberry pomace was obtained from the factory (Zhejiang Juxianzhuang Beverage Co., Ltd., Taizhou, China) and stored at −20 °C until use.

The commercial yeast and lactic acid bacteria powder were both purchased from the Angel Yeast (Shanghai, China). Acetic bacteria, bought from supplier (Shanghai Difa Brewage Biology Products Co., Ltd., Shanghai, China), was evenly mixed with bran. All bacterial powders were activated in accordance with the instructions of the manufacturer. The bacteria powders stored at −20 °C were left to stand at room temperature for 30 min, and then 0.1 g of bacteria powder were weighed and dissolved in 0.5 mL of distilled water and activated at 28 °C for 30 min.

### 2.3. Fermentation of Chinese Bayberry Pomace Wine

The unfermented sample was set up as control sample 1 (CS1). The Chinese bayberry pomace, inoculated with 0.02% yeast and fermented at 25 °C for 6 d, was set up as control sample 2 (CS2). The Chinese bayberry pomace wine was fermented according to our previous methods [3]. The Chinese bayberry pomace wine was vacuum-concentrated to 1/10 of its original volume at 55 °C. The remaining liquid was centrifuged to remove the sediment. The supernatant was vacuum freeze-dried to obtain CPW powder.

### 2.4. Antioxidant Capacity Analysis

The CPW diluted properly with water was used to evaluate the antioxidant activity by three classical chemical-based antioxidant capacity assays. Vitamin C (Vc) and trolox were used for calibration in all assays, DPPH and ABTS values were expressed as μmol Vc Equivalents per liter of sample (μmol Vc Eq/L), and FRAP value was expressed as mmol Trolox Equivalents per liter of sample (mmol Trolox Eq/L). 

The DPPH, ABTS, and FRAP scavenging activities of CPW were determined by our previous method [21]. In DPPH assay, CPW was diluted 1:20 with water, and then 2 mL of dilutions were mixed with 4 mL DPPH working solution (0.2 mM, in methanol). Deionized water was used as the blank control. Reaction solution was added to 96-well plates in sequence, and the mixture was left at room temperature in the dark for 30 min. Absorbance values were measured at 517 nm. In ABTS assay, 20 μL CPW was added to 180 μL of ABTS solution (0.2 mM, dissolved with 20 mg potassium persulfate in PBS buffer), and the mixture was placed in the dark at 30 ℃ for 6 min. Distilled water was used as the blank control. Absorbance values were measured at 734 nm. The FRAP assay was performed using a FRAP Kit. The 100 mM FeSO_4_ solution was diluted to 0.15 mM, 0.3 mM, 0.6 mM, 0.9 mM, 1.2 mM, and 1.5 mM for standard curve preparation. Then, 5 μL of samples, 5 μL of distilled water (blank control), and 5 μL of FeSO_4_ standard solutions of various concentrations (standard curve) were added to 96-well plates containing 180 μL of FRAP working solution. Absorbance at 593 nm was measured after incubation at 37 °C for 3–5 min. The total antioxidant capacity of the samples was calculated from the standard curve.

### 2.5. Cell Culture

HepG2 cells, acquired from the cell bank of the Shanghai Institute Cell Biology, were cultivated in a complete DMEM medium (including 10% FBS and 1% penicillin-streptomycin) at 37 °C/5% CO_2_ in a cell culture dish (Corning Incorporated). The cells were treated with trypsin, and they were grown to 80–90% confluence. The cells were then resuspended for further passage or CPW treatment. 

### 2.6. H_2_O_2_-Injured Cell Model (HI) and CPW Treatment

The H_2_O_2_-injured cell model (HI) was established according to the previous study with some modifications [22]. The trypsin-treated cells were seeded in the 24-well plates at a density of 2 × 10^5^ cells per well and cultured for 24 h. The medium was then replaced with DMEM containing H_2_O_2_ (0–1000 μmol/L) for 30 min to establish H_2_O_2-_induced HepG2 cell model. As a control, the medium with normal HepG2 cells was replaced by DMEM without H_2_O_2_. In the CPW-treatment group, the normal HepG2 cells were pre-incubated with CPW at different concentrations (20–600 μg/mL) for 4 h, then the medium was replaced with DMEM containing H_2_O_2_ (50 μmol/L) for 30 min. Afterward, all groups were cultured for another 24 h in a complete medium for the following assays.

### 2.7. Cytotoxicity Assay

The effect of CPW on the cell viability of normal HepG2 cells and H_2_O_2_-induced HepG2 cells was conducted by the MTT method [23]. Simply put, 100 μL of cells at the concentration of 1 × 10^5^ cells/mL were inoculated in 96-well plates and cultured in a 37 °C/5 % CO_2_ incubator (STIK instrument equipment (Shanghai) Co., Ltd., Shanghai, China) for 24 h. Afterward, the cell was washed with PBS 3 times and resuspended in culture medium with 100 μL CPW at different concentrations for another 24 h incubation. Then, the medium was discarded and replaced with 20 μL of MTT solution. After a 4 h incubation, 150 μL of DMSO was added after washing cells (using PBS). Absorbance at 570 nm was recorded after 10 min of oscillation, and cell viability was calculated after subtraction of a blank (DMSO only) from the absorbance obtained at 570 nm.

### 2.8. Intracellular ROS Determination

Intracellular ROS was detected using a fluorogenic dye DCFH-DA [24]. HepG2 cells were seeded in a 96-well plate at a density of 1 × 10^5^ cells/mL. After the treatment described above, the cells were washed with cold PBS and were further dark-incubated at 37 °C in 10 μM DCFH-DA for 20 min. Lastly, the cells were washed with cold PBS and then observed immediately with a Hitachi F-4500 fluorescence spectrophotometer (Hitachi, Tokyo, Japan) and a laser scanning confocal microscope (Leica TCS SP5, Wetzlar, Germany) at an excitation wavelength of 485 nm and an emission wavelength of 530 nm. Fluorescence values were calculated by subtracting a blank (medium only, no cells).

### 2.9. Cell Membrane Injury Determination

Cell membrane injury was detected using a fluorescent dye PI [25] with the final concentration of 50 μM. In 6-well plates, HepG2 cells at a density of 1 × 10^5^ cells/mL were cultured for 24 h. After washing with cold PBS twice, the cells were harvested at 37 °C and stained with 5 μL PI at a cold environment for 20 min. Lastly, a laser scanning confocal microscope (Leica TCS SP5, Wetzlar, Germany) was used to estimate cell membrane injury.

### 2.10. Intracellular Antioxidant Indexes Determination

HepG2 cells were inoculated in 6-well plates with or without CPW for 24 h. Then, the cells were cultured with 50 μM H_2_O_2_ for 6 h. Finally, the cells were lysed by RIPA buffer, centrifuged to obtain the cell supernatants, and kept at −20 °C. Afterward, the intracellular activities of SOD, CAT, and GR and the content of MDA, GSH, and GSSG of cell supernatants were detected by ELISA kits, and the results were adjusted to the protein concentration measured by a BCA protein assay kit.

### 2.11. RT-qPCR Analysis of mRNA Expression

The total RNA was isolated by TRIGene reagent from the cells. The quality and quantity of RNA were checked by NanoDrop spectrophotometer (Thermo Fisher Scientific, Waltham, MA, USA). Reverse transcription was achieved from total RNA with PrimeScript™ RT reagent kit. The mRNA expression level was determined by Real-time qPCR on a Quant Studio 3 Real-Time PCR System (Applied Biosystems, Foster, CA, USA) with Power SYBR green master mix. GAPDH were normalized as control and standardized as the expression level of 1.0. The results were expressed as the fold change in transcription level over the controls using 2^−ΔΔCt^ equation. Table 1 showed the primer sequences of mRNA.

### 2.12. Statistical Analysis

The data were reported as mean (*n* = 3) ± standard deviation (means ± SD) in triplicate. Statistical significance and mean differences between groups were analyzed by one-way ANOVA and Duncan’s test (*p* < 0.05) in SPSS version 22.0 (SPSS Statistics, IBM, Chicago, IL, USA).

## 3. Results and Discussion

### 3.1. Analysis of Antioxidant Activity

Fruit wine contains phenols, exopolysaccharides, enzymes, vitamins, and other substances and has the ability to remove free radicals. In order to assess the antioxidant activity of CPW, DPPH, ABTS, and FRAP assays were all employed.

**Figure 1:**
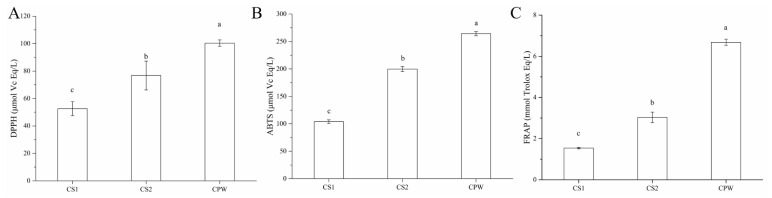
The antioxidant ability of CS1, CS2, and CPW, determined by DPPH (**A**), ABTS (**B**), and FRAP (**C**); DPPH and ABTS values were expressed as μmol Vc Eq/L, and FRAP value was expressed as mmol Trolox Eq/L. CS1: unfermented sample; CS2: fermented sample of Chinese bayberry pomace using yeast; CPW: mixed fermented sample of Chinese bayberry pomace using yeast, lactic acid bacteria, and ethylic acid bacteria; Vc: Vitamin C; Eq: Equivalents. Data are shown as mean ± SD (*n* = 3). Means in each column with different letters represent significant differences (*p* < 0.05).

As shown in Figure 1A, the DPPH value of CPW was 100.35 μmol vitamin C (Vc) Eq/L, which was 90.84% higher than that of CS1 (50.58%) and 30.73% higher than that of CS2 (76.77%). This indicated that the co-fermentation of Chinese bayberry pomace produced into wine could significantly improve its DPPH-scavenging ability. As shown in Figure 1B, the value of CPW was 264.20 μmol Vc Eq/L, which was 154.37% higher than that of CS1 and 32.19% higher than that of CS2. The results were consistent with the DPPH clearance rate, indicating that a mixed fermentation of Chinese bayberry pomace could also significantly improve its ABTS clearance ability. As presented in Figure 1C, the FRAP antioxidant activity of CPW was 6.67 mmol Trolox Eq/L, which was 333.12% higher than that of CS1 (1.54 mmol Trolox Eq/L) and 120.13% higher than CS2 (3.03 mmol Trolox Eq/L). The results were consistent with the results of DPPH and ABTS. In short, a mixed fermented Chinese bayberry pomace could improve the antioxidant capacity of wine. These results were in agreement with studies on Chinese bayberry pomace exhibiting high antioxidant activity after the fermentation of mixed probiotics [21]. Since the antioxidant activity of CPW in vitro is considerable, we were expected to verify its antioxidant function in a HepG2 cell model.

### 3.2. CPW Ameliorated the Cytotoxicity of H_2_O_2_-Induced HepG2 Cells

**Figure 2:**
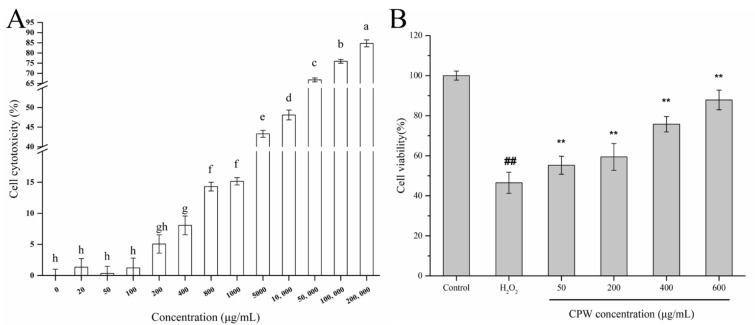
(**A**) The cytotoxic effects of CPW on HepG2 cells. Data are shown as mean ± SD (*n* = 3). Means in each column with different letters represent significant differences (*p* < 0.05). (**B**) Proliferative effects of CPW on H_2_O_2_-induced HepG2 cells. Control: normal HepG2 cells; H_2_O_2_: H_2_O_2_-injured cell model (HI); CPW: H_2_O_2_-injured HepG2 cells pretreated with CPW (50–600 μg/mL). ## *p* < 0.01 vs. control group; ** *p* < 0.01 vs. HI group.

In the MTT assay, CPW with different concentrations in the range of 20–200 μg/mL had no significant cytotoxicity on HepG2 cells (*p* > 0.05, Figure 2A). The cytotoxic levels of HepG2 cells treated with 200 to 1000 μg/mL CPW were all less than 15%. However, the cytotoxic levels increased sharply with a further increase in dose. CPW treatment was safe for cells if the cell cytotoxicity was lower than 15%. Therefore, CPW with the concentration of 50–600 μg/mL was selected for subsequent experiments.

H_2_O_2_ is often used as ROS to induce the oxidative injury of HepG2 cells. To examine the effect of CPW on the proliferation of H_2_O_2_-induced HepG2 cells, the cells were preincubated with CPW for 4 h with 50 μM H_2_O_2_ for 0.5 h. As shown in Figure 2B, the cell viability of HepG2 cells treated with H_2_O_2_ alone was markedly decreased (46.49%) compared with the control (*p* < 0.01), which indicated that the H_2_O_2_-injured cell model (HI) was successfully constructed. In comparison with the HI group, cell viability increased remarkably when pretreated with CPW before H_2_O_2_ induction (*p* < 0.01). The protective effect was more obvious as the concentration of CPW increased from 50 μg/mL to 600 μg/mL, showing a striking dose-–response relationship. Additionally, the concentration of CPW at 600 μg/mL led to the strongest repression effect against the H_2_O_2_-induced injury, with cell viability values 41.36% higher than those of the HI group. The data indicated that CPW had the potential to encourage a protective effect vs. the H_2_O_2_-induced injury in HepG2 cells. A recent study showed that *Musella lasiocarpa* fermentation broth had a certain effect to reduce the oxidative damage of HepG2 cells induced by CH_3_CH_2_OH and CCl_4_, which was similar to the results of our research [26]. The Chinese bayberry is rich in amino acids, phenolics, flavonoids, and other substances, with highly nutritional and antioxidant effects [27]. After fermentation, the content of phenolic compounds (Table S1) and polysaccharides was obviously increased, which led to the above phenomenon [2,3]. The concentration of CPW at 600 μg/mL, which had the strongest antioxidant activity, probably contained more active ingredients. Thus, the protective effects of the CPW may be attributed to the antioxidant activity based on the activity of free radical scavenging and the reduced power of ingredients such as anthocyanins, whereby the toxicity and the change in the antioxidant state of the damaged HepG2 cells were regulated. Therefore, CPW has the potential to be a natural health drink.

### 3.3. CPW Decreased ROS Production in H_2_O_2_-Induced HepG2 Cells

**Figure 3:**
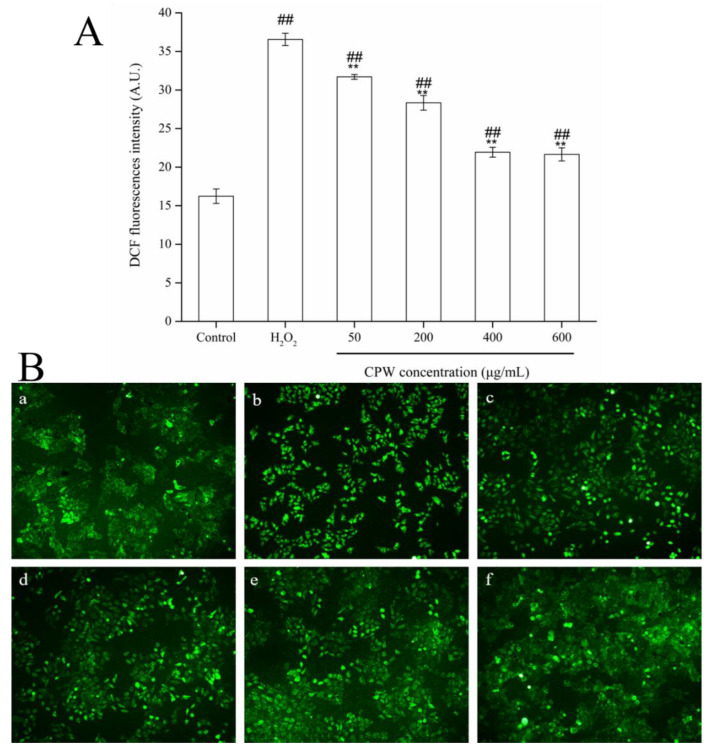
Effect of CPW on the intracellular reactive oxygen species (ROS) level in H_2_O_2_-induced HepG2 cells. Cells stained by DCFH-DA were measured by a fluorescence spectrophotometer (**A**) and observed under a laser scanning confocal microscope (**B**). Control: normal HepG2 cells; H_2_O_2_: H_2_O_2_-injured cell model (HI); CPW: H_2_O_2_-injured HepG2 cells pretreated with CPW (50–600 μg/mL). Data are shown as mean ± SD (*n* = 3); ** *p* < 0.01 vs. control group; ## *p* < 0.01 vs. HI group. (**a**). Control, (**b**). HI, (**c**). HI with 50 mg/L of CPW, (**d**). HI with 200 mg/L of CPW, (**e**). HI with 400 mg/L of CPW, and (**f**). HI with 600 mg/L of CPW (magnification, 100×).

In order to ascertain whether the protective effect of CPW on cells is associated with its antioxidant activity, a DCFH-DA fluorescent probe was used to evaluate the ROS level in cells. Intracellular esterase could hydrolyze DCFH-DA to DCFH, and then DCFH was oxidized to fluorescent DCF by intracellular ROS. Antioxidants inhibited the oxidation of DCFH and decreased the generation of DCF. As shown in Figure 3A, the fluorescence intensity of the control group was 16.23, whereas the fluorescence intensity of the HI group was 36.56, indicating that the intracellular ROS level in the cells increased significantly after H_2_O_2_ treatment (*p* < 0.01). Additionally, the DCF fluorescent signal released from cells in each CPW group was considerably lower than that in the HI group (*p* < 0.01); the higher the CPW concentration is, the weaker the DCF fluorescence intensity is. The images of the HepG2 cells processed with CPW demonstrated the same result (Figure 3B). The results showed that CPW has a good inhibitory effect on ROS formation in HepG2 cells under oxidative damage conditions and mitigates the oxidative damage of ROS in HepG2 cells, giving evidence that the cytoprotection of CPW may be related to its antioxidant capacities. Likewise, CPW at the concentration of 600 μg/mL minimized the fluorescence intensity of H_2_O_2_-induced HepG2 cells, indicating the supreme scavenging activity of ROS in cells. These results supported the claim that CPW has strong antioxidant properties and can effectively mitigate H_2_O_2_-induced oxidative damage.

On balance, a massive amount of H_2_O_2_ can accelerate the ROS accumulation in HepG2 cells and conduce the imbalance in ROS generation and cellular defense, leading to stress damage [24]. As previously reported, the high-molecular-weight polysaccharides produced during fermentation could enhance their oxidation resistance, which exerts a protective effect. It is expected that pretreatment with CPW alleviated these negative impacts by significantly reducing the accumulation of harmful intracellular ROS levels in the cells, which may provide protection against oxidative damage.

### 3.4. CPW Alleviated Cell Membrane Injury in H_2_O_2_-Induced HepG2 Cells

**Figure 4:**
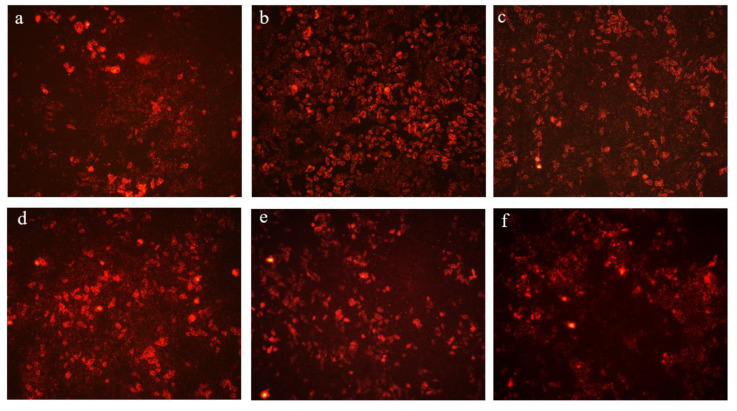
Protective effects of CPW against H_2_O_2_-induced membrane injury in HepG2 cells (magnification, 100×). (**a**) Control, (**b**) H_2_O_2_-injured cell model (HI), (**c**) HI with 50 mg/L of CPW, (**d**) HI with 200 mg/L of CPW, (**e**) HI with 400 mg/L of CPW, and (**f**) HI with 600 mg/L of CPW.

In the present study, HepG2 cells were treated to suffer anomalous apoptosis with 50 μM H_2_O_2_, and the level of cell membrane integrity was determined by the fluorescent dye PI, based on a laser scanning confocal microscope. PI has been widely used to stain apoptotic cells. PI can emit a red fluorescence if it penetrates the cell with a compromised membrane and binds to the nucleic acids [25]. The effects of different treatments on the changes in the membrane in HepG2 cells are shown in Figure 4. When HepG2 cells were treated with H_2_O_2_, a strong red fluorescence was induced in comparison with the control group. It is worth mentioning that pretreatment with CPW remarkably reversed this trend. Preincubation with CPW confirmed an essential protective impact on H_2_O_2_-induced apoptosis, so the percentage of apoptotic cells in the CPW group reduced significantly with the decreased number of red fluorescent cells compared with the HI group. Moreover, the effect of CPW in protecting HepG2 cells against H_2_O_2_-caused apoptosis was mainly because of their ability to clear more ROS in the cells. The excessive production of ROS disrupts the function of biological molecules, such as proteins and lipids, in cells, causing cell apoptosis [28]. These results were consistent with the ROS level described above. The results demonstrated that CPW has a certain capacity to improve membrane injury accompanied by a reduction in the oxidative damage caused by exogenous hydrogen peroxide.

### 3.5. CPW Counteracted the Oxidative Stress and Reinforced Antioxidant Capacity in H_2_O_2_-Induced HepG2 Cells

**Figure 5:**
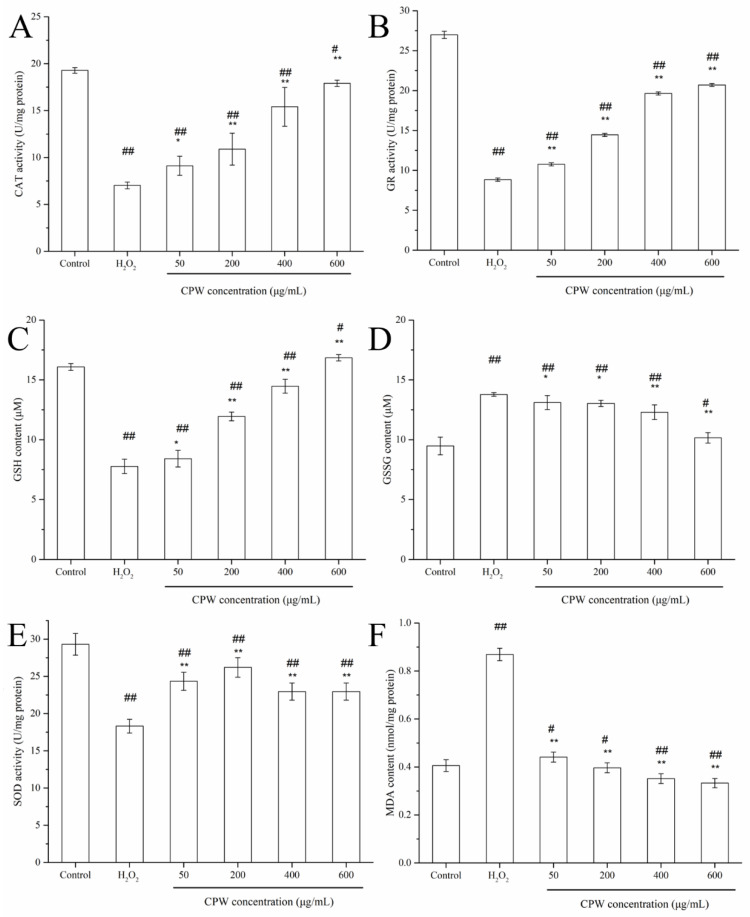
The effect of CPW on CAT (**A**), GR (**B**), GSH (**C**), GSSG (**D**), SOD (**E**), and MDA (**F**) levels in H_2_O_2_-treated HepG2 cells. Control: normal HepG2 cells; H_2_O_2_: H_2_O_2_-injured cell model (HI); CPW: H_2_O_2_-injured HepG2 cells pretreated with CPW (50–600 μg/mL). Data are shown as mean ± SD (*n* = 3); # *p* < 0.05 and ## *p* < 0.01 vs. control group; * *p* < 0.05 and ** *p* < 0.01 vs. HI group.

Intracellular ROS concentrations increased dramatically when H_2_O_2_ was added, resulting in oxidative stress in the cells. However, this injury could be prevented to some extent by the antioxidant system of the cells. A couple of antioxidant enzymes including SOD, CAT, and GR play a critical role in scavenging ROS and guarding against H_2_O_2_-induced injury. As shown in Figure 5, the H_2_O_2_-treated HepG2 cells displayed the decline of the intracellular CAT and GR levels by 63.52% and 67.20% (*p* < 0.01), respectively, while pretreatment of the cells with CPW markedly restored the decrease, and the activity increased to 17.91 ± 0.33 and 20.70 ± 0.19 U/mg protein, respectively. Moreover, this increase effect was dose-dependent, reaching a maximum recovery at 600 μg/mL. SOD activity also remarkably increased when the HepG2 cells were incubated with CPW compared with the HI group (*p* < 0.01). Nevertheless, the peak level of SOD appeared in the CPW group at 200 μg/mL, and then the enzyme activity decreased as the CPW concentration increased.

Glutathione (GSH) is the most abundant intracellular thiol-based antioxidant, which plays a crucial role in maintaining levels of ROS consistent with cellular function. Following oxidation by ROS, modification of the reduced GSH results in the formation of the oxidized GSSG. The GSH: GSSG balance is important in the regulation of pathways that are essential for maintaining homeostasis, normal cellular function, and overall survival. Meanwhile, the contents of GSH and GSSG and the GSH/GSSG ratio were also measured. In response to H_2_O_2_, the intracellular GSH content was decreased with a substantial increase in GSSG content, since the GSH/GSSG ratio was significantly lower. On the other hand, we observed a progressive increase in the GSH/GSSG ratio in HepG2 cells with CPW treatment. All the above data indicated that H_2_O_2_ induced a sharp transition from redox state toward intracellular oxidation, but CPW reversed the situation. A long-time oxidative condition may have meaningful impacts on cell function, as irreversible cell injury occurred when cells were no longer able to keep normal levels of GSH.

MDA, an indicator of the cellular lipid peroxidation, was significantly elevated two-fold in cells subjected to H_2_O_2_ stress when compared to the control (*p* < 0.01). Nevertheless, the addition of CPW had apparent inhibition on the production of MDA. When compared with the only group treated with H_2_O_2_, the concentration of CPW at 50 μg/mL, 200 μg/mL, 400 μg/mL, and 600 μg/mL reduced the content of MDA by 37.93%, 55.17%, 59.77%, and 62.07%, respectively.

Excessive ROS induced by H_2_O_2_ can cause measurably irreversible lesions in the microenvironment within the cells and ultimately cause cell apoptosis. These antioxidant enzymes stabilized or deactivated free radicals before assaulting the cellular components: superoxide was converted to H_2_O_2_ by SOD, and H_2_O_2_ was converted to H_2_O by CAT. GR is a flavoprotein that catalyzes the reduction of GSSG to GSH [29]. The enzyme is the main component of the cellular defense mechanisms against oxidative injury. Maintenance of a high GSH/GSSG ratio is important for the redox state of cells, and lowering this ratio usually indicates oxidative stress [30]. Bioactive peptides from the cartilage protein hydrolysate of *Mustelus griseus* could protect H_2_O_2_-caused HepG2 cells from oxidative damage by increasing the levels of SOD, CAT, GPx, and glutathione reductase (GSH-Rx) and decreasing the content of MDA [22]. Zhang also reported that fermented noni (*Morinda citrifolia* L.) fruit juice (FNJ) markedly ameliorated oxidative stress damage by activating or resisting related antioxidant enzymes activities such as SOD, GPx, and MDA in insulin-resistant HepG2 cells [31]. Endogenous antioxidation defense systems that comprise the antioxidation enzyme recovery system and the glutathione system play a pivotal role in the resistance to oxidative stress. These results strongly indicated that the pretreatment of cells with CPW can stimulate the cellular antioxidant system, accelerate the expression of antioxidative enzymes (SOD, CAT, and GR), and protect cells from H_2_O_2_-induced damage by scavenging ROS in the cells, which maintains the normal function of cells. In addition, the prevention of MDA generation indicated that CPW could inhibit lipid peroxidation, guarantee the integrity of the membrane, and limit intracellular ROS accumulation, contributing to the antioxidant properties.

### 3.6. CPW Regulated the Expression of Antioxidant Related Genes

**Figure 6:**
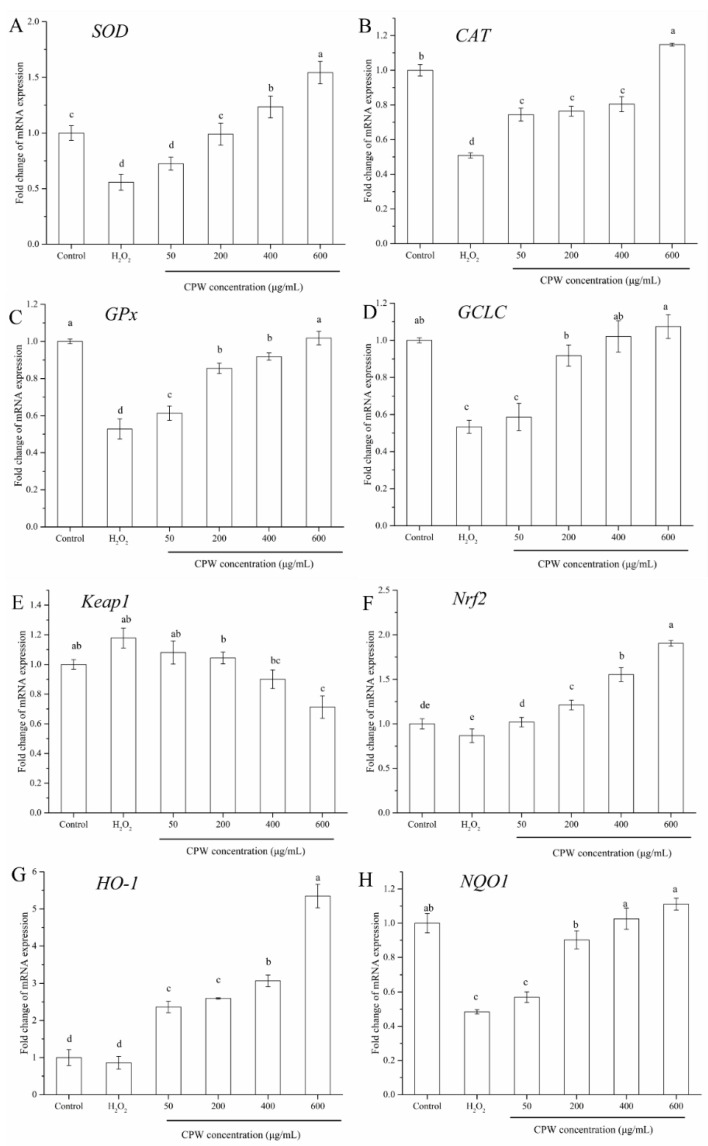
The effect of CPW on the expression of antioxidant genes [SOD (**A**); CAT (**B**); GPx (**C**); GCLC (**D**); Keap1 (**E**); Nrf2 (**F**); HO-1 (**G**) NQO1 (**H**)] in H_2_O_2_-injured HepG2 cells. Control: normal HepG2 cells; H_2_O_2_: H_2_O_2_-injured cell model (HI); CPW: H_2_O_2_-injured HepG2 cells pretreated with CPW (50–600 μg/mL). Data are shown as mean ± SD (*n* = 3). Means in each column with different letters represent significant differences (*p* < 0.05).

Oxidation-associated genes were detected by RT-qPCR after cultivation for 2 days. By comparison with the HI group, the expressions of the *SOD*, *CAT*, *GSH*, and *GCLC* genes were notably improved by CPW (*p* < 0.05). CPW at the concentration of 600 μg/mL showed the best performance in regards to the upregulation of the expressions of the *SOD*, *CAT*, *GSH*, and *GCLC* genes. CPW had a particular influence on the transcription of Keap1/Nrf2 pathway-associated genes (*Keap1, Nrf2, HO-1,* and *NQO1*). The expressions of *HO-1* and *NQO1* in the CPW and control groups all exceeded those of the HI group. Moreover, the expression of *Keap1* was downregulated, especially in the CPW group compared to the HI group, while the mRNA expression of *Nrf2* increased, especially in the CPW group compared to the HI group (*p* < 0.05; Figure 6). These results suggested that CPW may regulate the oxidative metabolism by means of the Keap1/Nrf2 signaling pathway. As a consequence, we found that the transcription of Nrf2 was notably increased and that Keap1 was markedly decreased by CPW (Figure 6).

GCLC is part of the glutamate cysteine ligase (GCL) that limits the synthesis rate of GSH and is modulated by the Nrf2/ARE signaling pathway. GCLC is frequently used as an indicator to activate the Nrf2/ARE pathway. Novel structure compounds (WS) containing 3,4,5-trimethoxyphenyl and acyl pyrazole moieties exhibit desirable protection against H_2_O_2_-caused PC12 cell injury by activating the Nrf2 pathway and upregulating the mRNA transcription of GCLC and protein expression [32]. HO-1 is a rate-limiting enzyme in heme catabolism, which participates in and regulates the oxidative stress response of the organism. Hyperactivation of Nrf2 and the subsequent overexpression of HO-1 contribute to tumor growth and tumor deterioration as well as resistance to anticancer therapy [33].

**Figure 7:**
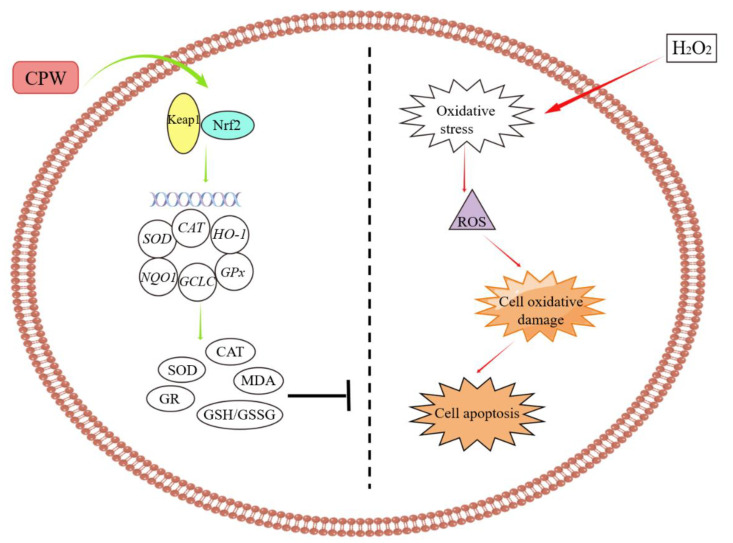
Cellular antioxidant mechanism of the CPW in H_2_O_2_-induced HepG2 cell.

Nrf2, a transcription factor, is a key player in the corresponding defense responses of cells by regulating the elementary and inducible expressions of many cytoprotective genes [34]. Under natural circumstances, Nrf2 is inactivated by binding to Keap1, which is able to sense oxidation/electrophilic signals [35]. Under oxidative stress, when Keap1 is degraded and deactivated, Nrf2 is released and is late-transferred to the cell nucleus, which initiates the expression of downstream antioxidant enzymes and, thus, restores cellular redox homeostasis. These results revealed that CPW can activate the Keap1/Nrf2 signaling pathway to supply antioxidants that protect the cell from oxidative stress. The cellular antioxidant mechanism is shown in Figure 7. This research was consistent with a report stating that the Keap1/Nrf2 signaling pathway was crucial for reducing oxidative injury [36,37].

## 4. Conclusions

This study found that Chinese bayberry pomace wine was capable of antioxidant activity, which was confirmed by chemical antioxidant evaluation methods and a H_2_O_2_-induced cell damage model. CPW exhibited protection against H_2_O_2_-induced HepG2 cell damage in a concentration-dependent manner. The mechanism study demonstrated that by activating the Keap1/Nrf2 signaling pathway and promoting the expression of intracellular antioxidant enzymes, CPW eliminates ROS and prevents the fragile cell membranes from being oxidized, thereby ensuring the steady state of the cellular microenvironment and inhibiting cell death. The increased antioxidant activity of CPW was dose-dependent, and CPW at the concentration of 600 μg/mL showed the most advanced protective effect against H_2_O_2_-induced cytotoxicity. Therefore, the technology studied here could be potentially applied to functional food production. Nevertheless, future studies should focus on the antioxidant activity of CPW in vivo and whether it is toxic or pathologically harmful in animal experiments.

## Figures and Tables

**Table 1:** The primer sequences of target genes for qPCR.

Gene	Forward Primer Sequences (5′-3′)	Reverse Primer Sequences (5′-3′)
*GAPDH*	CGACCACTTTGTCAAGCTCA	AGGGGTCTACATGGCAACTG
*SOD*	TGGAGATAATACAGCAGGCT	AGTCACATTGCCCAAGTCTC
*CAT*	CCATTATAAGACTGACCAGGGC	AGTCCAGGAGGGGTACTTTCC
*Nrf2*	AGTGTGGAGAGGTATGAGCC	CGTTCCTCTCTGGGTAGTAA
*Keap1*	AGAGCGGGATGAGTGGCA	GCTGAATTAAGGCGGTTTGTC
*NQO1*	GAAAGGATGGGAGGTGGTGG	CTGGAGTGTGCCCAATGCTA
*HO-1*	ATCGCTACTTCCCTGCCTTTG	GAGGGAAGCTGGAAATAAGGCTA
*GCLC*	TGGGCAATTGCTGTCTCCAG	TCGCTCCTCCCGAGTTCTA
*GPx*	AGAAGTGCGAGGTGAACGGT	CCCACCAGGAACTTCTCAAA

## Data Availability

Data will be made available on request.

## Supplementary Materials

The following supporting information can be downloaded at: https://www.mdpi.com/article/10.3390/foods12091863/s1. Table S1: Content of phenols and flavonoids in test samples [2,38,39].
