# Supplementary material for: Protective Effect of Chinese Bayberry (Myrica rubra Sieb. et Zucc.) Pomace Wine on Oxidative Stress of Hydrogen Peroxide by Regulating Keap1/Nrf2 Pathway in HepG2 Cells
Source: Foods. 2023 Apr 30;12(9):1863. doi: 10.3390/foods12091863 (PMC10178721; doi:10.3390/foods12091863)
Supplement: Supplementary file 1 [file foods-12-01863-s001.zip › foods-2310418-supplementary.pdf]

Supplementary material

Determination of total phenolic content (TPC) and total flavonoids content (TFC)

The content of the total phenolics was measured using the Folin–Ciocalteu method [1]. The sample (50 μL) was diluted with 450 μL water and then oxidized with 2.5 mL of Folin–Ciocalteu reagent in the dark for 3 min at room temperature, followed by the addition of 2 mL of 7.5% Na_2CO_3. After 60 min of incubation at room temperature in the dark, the absorbance of the resulting blue color was measured at 765 nm. The total phenolics were calculated from the standard curve (0-500 mg/L) for gallic acid.

The content of the total flavonoids was determined according to the method reported by Zhang et al. [2], with slight modification. Briefly, the samples were diluted 1:10 with water, then 50 μL of dilutions was added to a 1.5 mL tube containing 75 μL of 5% NaNO2 solution. After 5 min, 150 μL of AlCl3 solution (10%) was added to react for 6 min. Finally, 500 μL of 1 mol/L NaOH was added into the reaction system, and 1275 μL water was used to increase the volume to 2.5 mL. After 10 min, the absorbance was determined at 510 nm. The total flavonoids were calculated from the standard curve (0-500 mg/L) for rutin.

HPLC analysis of phenolic acids and flavonoids

The analysis was carried out by means of an HPLC system equipped with a Waters e2695 Separations Module, a Waters 2998 UV/Visible Detector, and Empower software. The chromatographic column was a 4.6 × 250 mm, 5 μm, Green Mall GU-C18 column. The column temperature was maintained at 30 ◦C. The mobile phases consisted of 2% acetic acid with acetonitrile (A) and 2% aqueous acetic acid (B). The flow rate was 0.8 mL/min. The gradient program was as follows: 0–60 min, 5%– 15% A; 60–65 min, 15% A; 65–66 min, 15%–20% A; 66–73 min, 20% A; 73–74 min, 20%–30% A; 74–80 min, 30% A; 80–81 min, 30%–40% A; 81–93 min, 40% A; 93–95 min, 40-5% A. The injection volume was 20 μL, and the detection wavelengths were 280 nm, 320 nm, and 520 nm.

Table S1. Content of phenols and flavonoids in test samples [3].

Samples	CS1	CS2	CPW
Gallic acid (mg/L)	12.84 ± 0.04	124.35 ± 0.39	156.51 ± 0.14
Protocatechuic acid (mg/L)	29.72 ± 0.06	52.40 ± 0.12	72.90 ± 0.07
Myricitrin (mg/L)	244.64 ± 0.02	217.25 ± 0.09	275.36 ± 0.07
Isoquercitrin (mg/L)	3.15 ± 0.02	3.62 ± 0.06	8.63 ± 0.03
Quercitrin (mg/L)	120.12 ± 0.13	102.23 ± 0.09	130.62 ± 0.11
Myricetin (mg/L)	-	-	10.37 ± 0.01
Quercetin (mg/L)	-	-	5.38 ± 0.01
Anthocyanin (mg/L)	113.51 ± 1.19	31.63 ± 0.41	50.18 ± 3.17
Total phenols (mg/L)	1082.25 ± 4.34	1662.42 ± 10.59	2092.75 ± 30.97
Total flavonoids (mg/L)	312.05 ± 1.91	462.82 ± 4.53	666.41 ± 6.89

Note: Values represent means of triplicate determination ± SD.

References

1. Tian, J.; Chen, J.; Lv, F.; Chen, S.; Chen, J.; Liu, D.; Ye, X. Domestic cooking methods affect the phytochemical composition and antioxidant activity of purple-fleshed potatoes. *Food Chem*. **2016**, *197*, 1264-1270.
2. Zhang, Z.; Li, J.; Fan, L. Evaluation of the composition of Chinese bayberry wine and its effects

on the color changes during storage. *Food Chem*. **2019**, *276*, 451-457.

3. Zhu, Y.; Lv, J.; Gu, Y.; He, Y.; Chen, J.; Ye, X.; Zhou, Z. Mixed fermentation of Chinese bayberry pomace using yeast, lactic acid bacteria and acetic acid bacteria: Effects on color, phenolics and antioxidant ingredients. *Lwt* **2022,** *163*, 113503.
